# A novel isatin Schiff based cerium complex: synthesis, characterization, antimicrobial activity and molecular docking studies

**DOI:** 10.1186/s12864-024-10037-3

**Published:** 2024-02-08

**Authors:** Heba E. Saad, Gaber M. Abu El-Reash, Mohamed gaber, Mohamed A. Hashem, Yasmeen G. Abou El-Reash, Nuha Y. Elamin, Mohamed R. Elamin, Yusif S. El-Sayed

**Affiliations:** 1 Department of Chemistry, Faculty of Science, Tanta University, Tanta, 31527 Egypt; 2 Department of Chemistry, Faculty of Science, Mansoura University, Mansoura, 35516 Egypt; 3 Department of Chemistry, College of Science, Imam Mohammad Ibn Saud Islamic University (IMSIU), P.O. Box, 90950, 11623 Riyadh, Saudi Arabia; 4 Department of Chemistry, Sudan University of Science and Technology, P.O. Box 407, Khartoum, 11111 Sudan

**Keywords:** Isatin-Schiff base L2, Antimicrobial activity, Cerium (III)-Schiff base complex C2, Molecular operating environment (MOE), TGA and hydrothermal reaction

## Abstract

In this work, a novel isatin-Schiff base L2 had been synthesized through a simple reaction between isatin and 2-amino-5-methylthio-1,3,4-thiadiazole. The produced Schiff base L2 was then subjected to a hydrothermal reaction with cerium chloride to produce the cerium (III)-Schiff base complex C2. Several spectroscopic methods, including mass spectra, FT-IR, elemental analysis, UV–vis, ^13^C-NMR, ^1^H-NMR, Thermogravimetric Analysis, HR-TEM, and FE-SEM/EDX, were used to completely characterize the produced L2 and C2. A computer simulation was performed using the MOE software program to find out the probable biological resistance of studied compounds against the proteins in some types of bacteria or fungi. To investigate the interaction between the ligand and its complex, we conducted molecular docking simulations using the molecular operating environment (MOE). The docking simulation findings revealed that the complex displayed greater efficacy and demonstrated a stronger affinity for Avr2 effector protein from the fungal plant pathogen Fusarium oxysporum (code 5OD4) than the original ligand. The antibacterial activity of the ligand and its Ce^3+^ complex were applied in vitro tests against different microorganism. The study showed that the complex was found to be more effective than the ligand.

**Supplementary Information:**

The online version contains supplementary material available at 10.1186/s12864-024-10037-3.

## Introduction

Some Di and trivalent lanthanide complexes (i.e. Sm^2+^, Ce^3+^ and Eu^2+^) have many promising luminescence applications and biological activities [1, 2]. These ions are characterized by their 5d – 4f electron transitions, which is spin- and parity-permissible, leading to nanosecond fluorescent durations. Cerium is characterized by an electronic configuration of 4f^1^ 5d^0^ in its ground state. The exposed 5d orbitals are more prone to the influence of ligand fields (LFs) compared to the 4f orbitals that are protected by the surrounding 5s and 5p. The 5d orbitals undergo variable degrees of splitting when subjected to different LFs interactions, so the energy gaps separating the 4f and 5d orbitals are altered by this mechanism. Therefore, the Ce^3+^ complexes can attain a complete-spectrum emission in theory. Because of its incomplete 4f subshell, cerium has a numerous magnetic, luminescence, optical and electronic properties [2, 3]. Cerium as ceria has several application such as catalyst [4], electrolyte material for SOFC [5, 6] sunscreen [7–9], gas sensors [10], fluorescent materials [11] and ceramic materials [12]. Cerium(III) complexes exhibit a fascinating luminescent phenomenon known as the doublet-doublet electron transition. This process involves the exchange of electrons between two doublet energy levels, from the first doublet excited state, ^2^D, to the doublet ground states, ^2^F_7/2_ and ^2^F_5/2_. The spectroscopic notation is derived from the Russell-Sanders formula, which serves as the basis for obtaining these notations ^2S+1^*L*_*J*_ (three quantum numbers: *S* = 1/2, *L* = 3, and *J* =|*L* ± *S*|= *5/2* or *7/2*).

The luminescence property of cerium complexes aren’t commonly seen from molecular cerium components but this property could be because of Cerium's excited state that is quickly quenched by a non-radiative relaxation passage [13, 14]. The ions exhibit a luminescent duration of a few milliseconds and the precise separation of the focused emission over time has greatly facilitated the advancement of imaging techniques. In light of this, we can utilize luminescent lanthanoid complexes as a probes to investigate and assess enzyme activities, optical imaging of glucose oxidase activities, in addition to the processes of tyrosine phosphorylation and dephosphorylation takes place in peptide biochemical reactions. Furthermore, complexes of cerium can function as an anticancer agent due to the planner conformation which stimulates them qualified to react with base pairs of DNA via intercalating method [15–17]. If we compared cerium to other metal complexes of lanthanide, cerium shows low toxicity. Thus, it could be effective to react with DNA in medicinal domains [18].

Isatin (1H-indole-2, 3-dione) and its derivatives such isatin-azole, isatin-quinoline, isatin-furan, coumarin, isatin-hydrazone, thiosemicarbazone, isatin-dimers and isatin-indole hybrids have a wide domain of pharmaceutical and biological property [19–22] and are quite used as a starter for synthesis of wide range of heterocyclic components and as substrates for synthesis of drugs [23]. Schiff bases compose a significant grade of organic components with a broad diversity biological properties [24–29], anti-malarial [30], anti-inflammatory [31], analgesic [32], and anti-anxiety [33], pharmaceutical properties like antiviral [34], antibacterial [35], anticancer [34], anticonvulsant activity, antidiabetic [31] and cytotoxicity antioxidant activity [36–42]. Isatin is a significant components of several drugs [43], alkaloids [44], pesticides, analytical reagents and dyes [45].

**Scheme 1:**
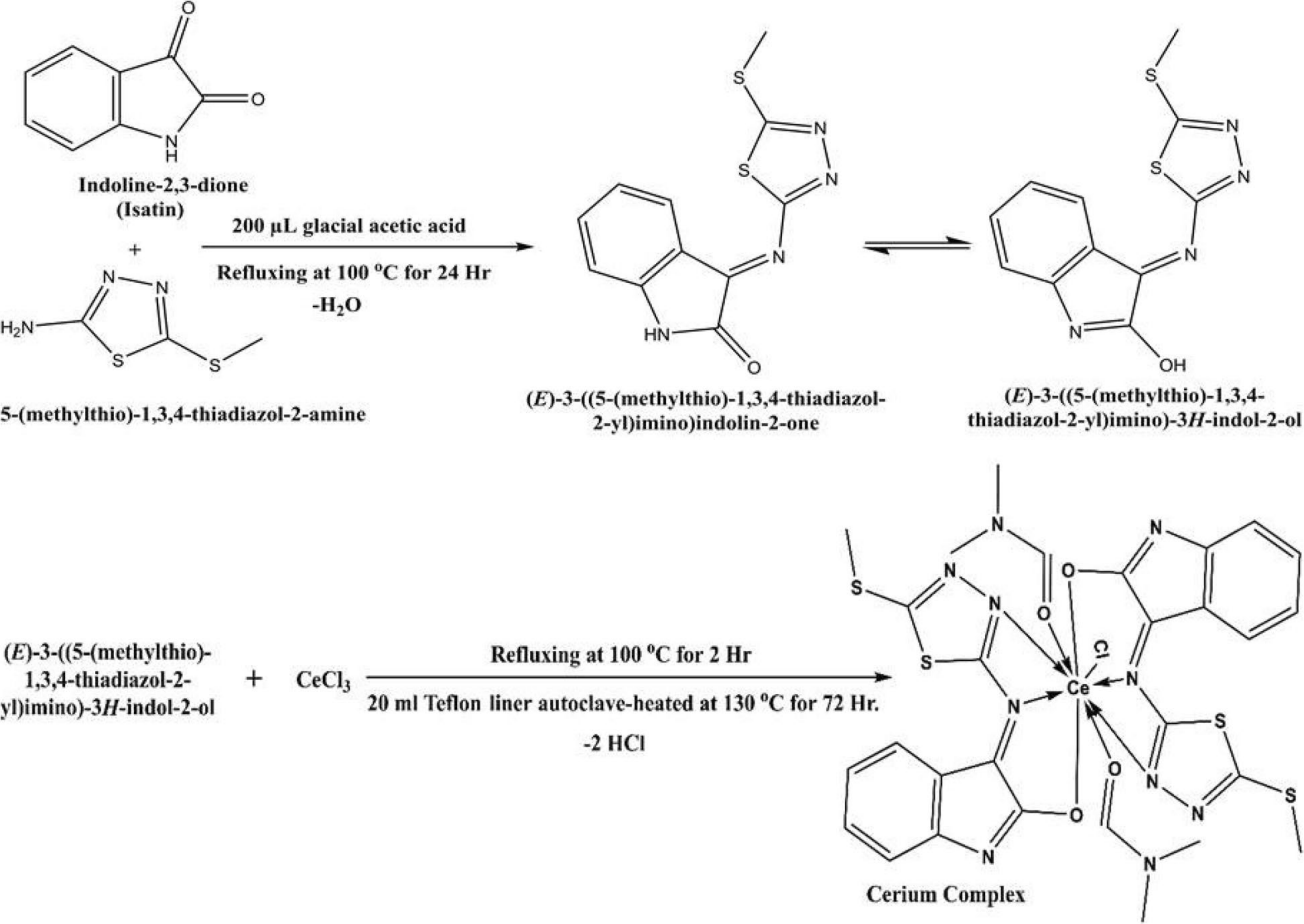
The proposed scheme of the reaction of Schiff base L2, and Ce(III)-complex C2

Significant advancements are currently being made in the field of luminescent cerium (III) complexes. Ce^3+^ Complexes possessing simple synthesis methods, excellent thermal stability, and a significantly high quantum yield in terms of photoluminescence are still quite rare. In this study, isatin was chosen as a ligand based on its exceptional biological properties that are mentioned previously to successfully synthesized a Ce3 + complex as shown in Scheme 1. The prepared complex was characterized by means of FT-IR spectral, Elemental analysis, XRD, TGA, DTG and molar conductance measurements. The obtained results pointed out that the ligand and its complex were less effective against the tested microorganisms when compared with the standard drugs. The study showed that the complex was found to be more effective compound than the ligand. Furthermore, the docking behavior of compounds had been carried out.

## Experimental and methods

### Materials

All chemicals and solvents which used in our research are of the analytical reagent grade and had been used as established. From Sigma-Aldrich, Isatin (C11H10), 2-amino-5-methylthio-1,3,4-thiadiazole (C_3_H_5_N_3_S_2_) and anhydrous cerium(III) chloride CeCl_3_; 99.9% had been obtained.

### Instruments

The characterization was conducted utilizing a variety of analytical tools: at room temperature, KBr tablets were used to record FT-IR spectra in the 400–4000 cm^−1^ range using the JASCO FT/IR-460 spectrophotometer (JASCO, USA). A JEOL JSM-6510LV advanced electron microscope with the LAB-6 cathode at 520 keV had been used to record the field emission scanning electron microscopy (FE-SEM) element and images produced via spatially determined energy dispersive X-ray spectroscopy (EDX) (JEOL, Japan). The produced metal complex and it’s Schiff base ligand (HL) were dissolved in ethyl alcohol for Ultra violet-visible (UV–vis) spectra that were recorded over a wavelength range of 200–900 nm in a 1 cm quartz cell Using the PerkinElmer 550 spectrophotometer. A high-resolution transmission electron microscope (HR-TEM) was used to examine the composition of produced phases with the acceleration voltage up to 200 kV (TEM, JEOL-JEM-2100, Tokyo, Japan). With the use of the Thermo Scientific, ISQ Single Quadrupole MS, the mass spectrum of the solid ligand and its complex had been recorded (Thermo Scientific, USA). The elemental map was tested on a Costech (ECS-4010) elemental analyzer from Costech, Italy, to determine the C, H, and N content. A Gemini-300 MHz NMR spectrometer was used to capture the 1H-NMR and 13C-NMR spectra in DMSO-d6 (ECA 500 II, JEOL, Japan). Thermal analysis (DTG/TGA) of the sample had been analyzed via using NETZSCH STA 409 C/CD, Germany with the rate of 10 °C min^−1^ in oxygen atmosphere. The samples have been evaluated at room temperature using a variety of excitement wavelengths, and Origin (9) software produced the analytical results.

### Methodology of synthesis of (E)-2-((5-methylthio)-1,3,4-thiadiazol-2-yl)imino)indolin-3-one (L2)

Schiff base have been prepared by dissolving 1.471 gm isatin in the following mixture (16 ml methanol/4 ml DMF) and then the mixture had been added to 2-amino-5-methylthio-1,3,4-thiadiazole (1.472 gm) dissolved in methanol (10 ml) in the presence of 200 µL glacial acetic acid. At 100 ºC the producing mixture had been refluxed for 24 h. The mixture had been left to cool, the color of the precipitated Schiff base was light brown (Scheme 1). The resulting precipitate had been dried in an oven at 100 ℃ after washing; the calculated yield was 97%.

### Methodology of synthesis of cerium (Ш) complex (C2)

According to the method described in, a (1.232 gm) of anhydrous cerium chloride CeCl_3_ had been dissolved in 10 ml of dist. water before being added to Schiff base (L2) as presented in (Scheme 1). This system had been heated to 130 °C in the autoclave for 72 h after being refluxed for two hours. The mixture was cooled to room temperature, and the precipitate has been dark brown (Scheme 1). The predicted yield was 90.4% after the collected precipitate was dried and washed with dist. water.

### Antimicrobial activity

The prepared compounds were assessed for their antimicrobial effects using the agar diffusion technique [46, 47]. A 1 mg/ml solution in dimethyl formamide (DMF) was used, while DMF alone served as a control with no observed inhibition zones. Bacteria and fungi were cultured on nutrient agar and Czapeks Dox agar medium, respectively, then were subjected to inoculation on agar media. Following 24 h of incubation at 30 °C for bacteria and 48 h at 28 °C for fungi, the diameter of the inhibition zone was measured in millimeters.

### Docking studies

Recently, the MOE software program proved to be a valuable tool for conducting detailed molecular docking studies. We accessed the protein data bank and given the prevalence of this protein in various bacterial species, we acquired the crystal structures with the assigned code 5OD4. All the water molecules surrounding the protein were removed, and hydrogen atoms were introduced instead. Each molecule that was analyzed underwent optimization using the MMFF94x force field to achieve its most favorable form with the lowest binding energy [48, 49]. The incredible MOE programmer's site finder was utilized to create the splendid Alpha-site spheres. The calculation of the free binding energy, which serves as an indicator of the strength of the bond, was derived from the acidic side chain donor alongside the metal ion contact between protein and the tested compounds. Through the process of docking the crystalline ligand and its metal chelate, we successfully validated our docking methodology and got root mean square deviation (RMSD) values 2.1349 and 3.292 kca/mol for ligand and its complex, respectively.

## Results and discussion

### Characterization of the Schiff base L2 and Ce (III)-complex C2

According to Scheme 1, the Ce(III) complex C2 and the Schiff base ligand L2 were easily synthesized in two different systems. The components L2 and C2 are both very colourful, soluble in both DMF and DMSO, non-hygroscopic, and constant in air. Table 1 presents the most crucial physical information for the synthesized components.

**Table 1:** Analytical and some important physical measurements for L2 and C2

**Assignments**		L2	**C2**
**Preparation Method**		Reflux	**Hydrothermal**
**Color**		Light brown	**Dark brown**
**Aspect**		Powder	**Fine crystal**
**Melting Point (°C)**		102	** > 300**
**Reaction Time**		1 days	**3 days**
**Yield (%)**		97	**90.4**
**Chemical Formula**		C_11_H_8_N_4_OS_2_	**C**_**28**_**H**_**28**_**CeClN**_**10**_**O**_**4**_**S**_**4**_
**Molecular Weight** (**g/mol)**		276.33	**872.40**
**Elemental analysis (Calc.)/ Found**	**C %**	(47.81)/ 46.95	**(38.54)/ 37.62**
	**H %**	(2.91)/ 2.54	**(3.23)/ 3.10**
	**N %**	(20.27)/ 20.11	**(16.05)/ 15.52**
	**S %**	(23.20)/ 23.09	**(14.69)/ 14.54**
**Characteristic infrared frequencies (cm**^**−1**^**)**	**ν(OH)**	3374	**3426**
	**ν(C = O)**	**–-**	**1813**
	**ν(C = N)**	1716	**1757**
	**ν(C-N)**	1473	**1380**
	**ν(C = C)**	1659	**1624**
	**ν(N–N)**	1195	**1127**
	**ν(C–S)**	754	**757**
	**ν(Ce–O)**	–-	**672**
	**ν(Ce–N)**	–-	**528**
**UV‐ λ**_**max**_** (nm)**		378, 397, 424, 452	**381, 415, 456, 631**

#### Elemental analysis

Table 1 shows the elemental analysis of both L2 and C2. The achieved results have been in approval with the theoretical calculations of the proposed formulations (%): for ligand L2; C_11_H_8_N_4_OS_2_; (276.33 g mol^−1^); C, 47.81; H, 2.91; N, 20.27; S, 23.20; found C, 46.95; H, 2.54; N, 20.11; S, 23.09. For the cerium(III)-complex C2; C_28_H_28_CeClN_10_O_4_S_4_; (872.40 g mol^−1^); C, 38.54; H, 3.23; N, 16.05; S, 14.69; found C, 37.62; H, 3.10; N, 15.52; S, 14.54.

#### FT-IR spectra

In Table 1 and Fig. S1, the most significant L2 and C2 frequencies in the FT-IR spectrum are displayed. There was an enough agreement between the two spectra. At 3374 cm^−1^, L2 has a large absorption band, that could refer to the ν(OH) group of the alcohol. The band at 1716 cm^−1^ is assigned to the ν(C** = **N) vibrations of the ring of (5-methylthio)-1,3,4-thiadiazol-2-amine. The two bands at 1659 and 1473 cm^−1^ correspond to the isatin moiety's v(C = C) and (C-N), individually. The band at 1195 cm^−1^ can be attributed to ν(N–N) group. The band at 754 cm^−1^ is assigned to ν(C–S) group [50]. Furthermore, the FT-IR spectra of C2 refers to the bond combination of the metal ion with the ligand. On the other hand, we observed a notable shift in the group of ν(OH), ν(C = N), ν(C = C), ν(C–N) and ν(N–N) vibrations in the spectrum of the free ligand L2 compared to that of C2. Meanwhile, the ν(C–S) vibration remained unchanged in its original position because this bond didn’t take a part in the coordination with the metal ion. The FTIR spectral analysis (Fig. S1) pointed out that L2 acted as a neutral bi-dentate coordinating ligand through ν(OH), (C–N) and azomethine nitrogen (C = N) atoms. In addition to this observation new bands were appeared in the 528 cm^−1^ and 672 regions attributed to ν(Ce–N) and ν(Ce–O), correspondingly [51].

#### UV–vis spectrum

Figure S2 displays the electronic absorption spectrum of the L2 and C2 in DMF at room temperature. From this figure, it can noticed that L2 displayed main four absorption bands at 378, 397, 424 and 452 nm, that referred to the transfer of charge inside the ligand (n–π* and π–π*). Meantime, C2 detects four absorption bands at 381, 415, 456 and 631 nm, that were referred to the probable intra ligand charge transfers (n–π* and π–π*) and ligand metal charge transfer transitions (LMCT) [52].

#### ^1^H-NMR spectrum

The ^1^H-NMR spectra of the ligand L2 Fig. S3 presented two signals at 12.57 ppm conformable to OH. The signals which noticed at the range of 6.5- 8.2 ppm could be explained by to the benzene ring protons. The signals at range 2.2–3 ppm are accredited to CH_3_ protons of methyl group. As shown in ^1^H-NMR spectra of the Ce-complex C2 (Fig. S4), the OH group exhibited a signal at 11.40 ppm. The protons of the benzene ring are responsible for the multiplied signals appeared in the range of 7–8.22 ppm, but the signals at 2.23 and 3.11 ppm comply with alcohol and CH_3_ of DMF solvent, respectively [53].

#### ^13^C-NMR spectra

The (C = O) and (C = N) groups of the phenyl ring are represented by two signals at 164.1 and 158.7 ppm in the ^13^C-NMR spectrum of L2 as shown in Fig. S5. Whereas the (C = C) in the benzene ring's groups are represented by the signals that were recorded between 150 and 120 ppm. The bands appeared in the range of 40–30 ppm corresponding to DMF and alcohol, but band which appeared at 18.7 ppm belongs to CH_3_ group. The ^13^C-NMR spectra of C2 complex present in Fig. S6 show signals at 169.8 and 154.7 ppm belongs to (C = N) and (C-O), groups of phenyl ring respectively. The band appears at 162.9 ppm is corresponding to carbonyl group of DMF solvent, but the signals at 149.5 to 124.7 ppm are attributable to carbons of benzene ring. Signals that appeared at 52 and 40 ppm are related to DMF and alcohol solvent, while signal at 19.8 ppm belongs to CH_3_ group.

#### Mass spectra

The electron influence mass spectrum of L2 and C2 have been registered and examined at 70 eV. The mass spectra and the suggested fragmentation of L2, respectively are demonstrated in Fig. S7 and Scheme S1. It has been renowned which the molecular ion peaks were in respected convention with their proposed experimental formulation as suggested from the elemental analysis. The ligand's exact mass is 276.62 and the theoretically estimations is 276.33 gmol^−1^ according to the formula C_11_H_8_N_4_OS_2_ proposed via the mass spectra fragmentation technique (Fig. S7). According to Scheme S1, the ion of m/z = 276.62 disintegrates to an established peak at m/z = 146.07 (supposed to be 146.15 gmol^−1^) by lost C_3_H_4_N_2_S_2_ atoms. The CS bond is broken, resulting in the fragment with m/z = 134.87. The CH_3_ atoms were lost, resulting in the fragment with m/z = 120.99. H_4_S_2_ atoms were lost, resulting in fragmentation with m/z = 55.17. The disintegration in Scheme S1 results from a fragmentation of L2. Additionally, the mass spectra and suggested breakdown of C2 were shown in Fig. S8 and Scheme S2, respectively. According to the formula C_28_H_28_CeClN_10_O_4_S_4_, the mass spectra fragment style of C2 shows an exact mass of 871.71 (theoretically planned as 872.40 gmol^−1^) (Fig. S8). According to Scheme S2, the ion at m/z = 871.71 suffers breakdown to a stabilized peak at m/z = 726.61 through lost two molecules of DMF. The fragment with m/z = 451.11 was created as a result of the losing of C_11_H_8_N_4_S_2_O atoms. The disintegration with m/z = 276.49 results from the loss of the (Ce – Cl) bond. C_3_H_4_N_2_S_2_ atoms were lost, resulting in the fragmentation with m/z = 146.63. Due to losing of CH_3_N atoms, disintegration occurred at m/z = 120.57, while when CH_3_NO ion are lost, the reaction breaks down with m/z = 78.10. The pieces in Scheme S2 are the result of the cerium complex's basic fragmentation. Most of the time, the sequent fragmentations of both L2 and C2 were improperly modified and exactly matched the molecular weight of the structures that were shown.

#### Thermo gravimetric analysis

The popular of complexes counting metal complexes can be different when heated in accordance with a specified experimental statement. The variations are be used for quantitative and qualitative exploration. We have two major of methods thermo-gravimetry (TGA) and the first derivative thermogravimetry (DTG). The weight of the sample varies as a result of numerous chemical changes (such as thermal breakdown, oxidation, etc.) and physical processes (such as solvent and water evaporation, sublimation, etc.) that occur when the sample to be studied is heated. The TGA method's goal is to examine this process.

The thermal degradation of Ce(III)-Schiff base complex C1 occur in 5 steps. The first step at [28.81–139.81] °C is corresponding to losing all DMF molecules with weight loss of 16.4% (Cal. 16.5%). Second step at [139.81–438.78 °C] is corresponding to the decomposition of C_11_H_8_N_4_S_2_O with weight loss 31.6% (Cal. 31.5%). The third step at [438.78–609.75] °C is corresponding to the weight loss of CeCl 19.95% (Cal. 20.01%). While the fourth step at [609.75–730.67 ℃] is referring to the decomposition of C_3_H_4_N_2_S_2_ with weight loss 15.08% (Cal. 15.02%). The fifth step at [730.67–921.17 ℃] confirm to the loss CH_3_N and CH_3_NO with the loss 3.02% and 5.25% (Cal. 3.17 and 5.1%), respectively and the residue of complex is C_6_H_6_ 8.69% (Cal. 8.7%) as shown in (Fig. S9 and Table S1).

##### FE-SEM/EDX

**Fig. 1:**
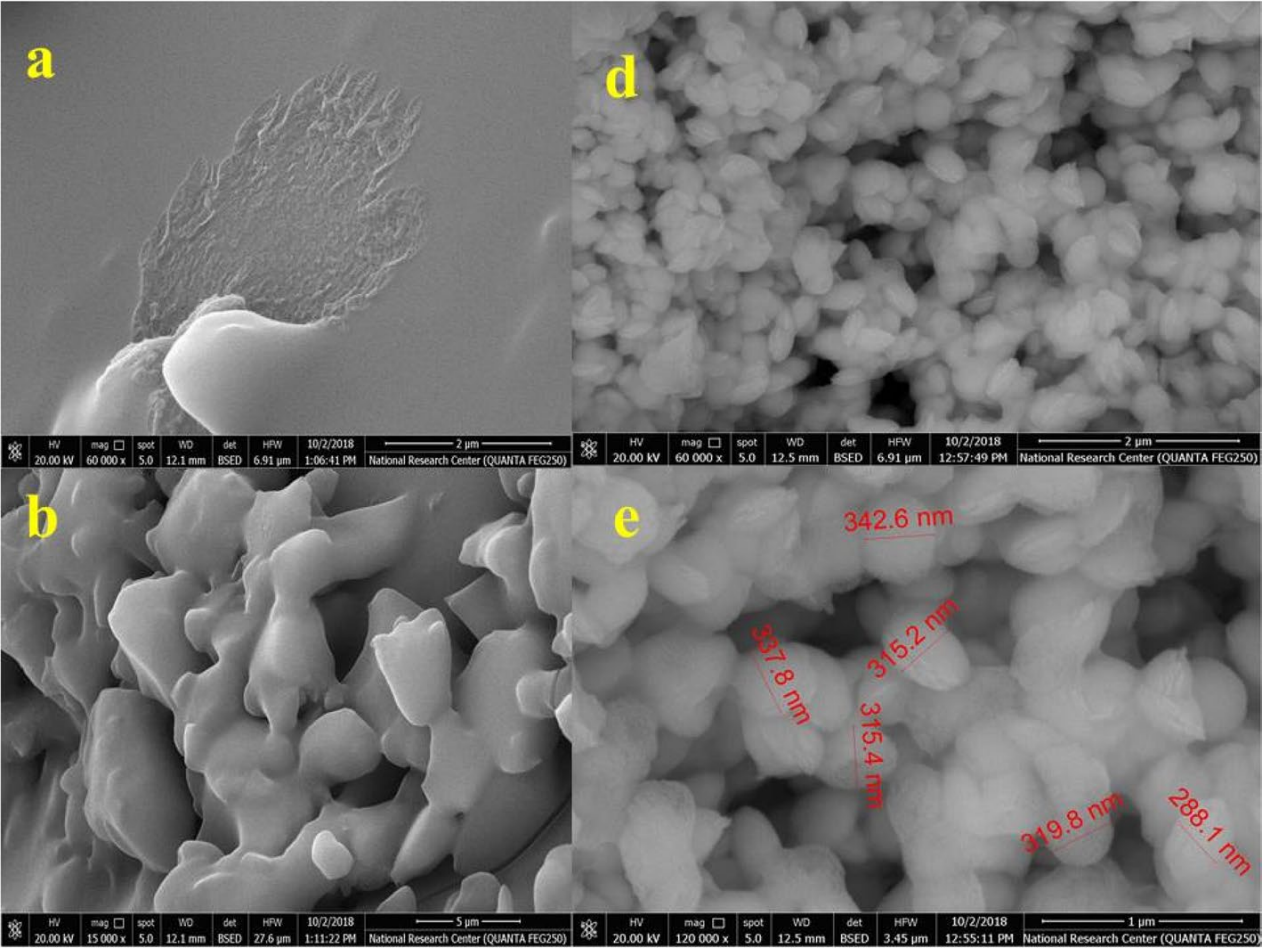
SEM images of [**a**–**b**] Schiff base L2, and [**d**–**e**] Ce(III)-complex **C**2

Figure 1 displays the FE-SEM imageries and EDX analysis of L2 and C2. While C2 in Figs. 1d and e appeared to depict a nanostructure oval particle form with a moderate size of 19.21 nm, L2 in Figs. 1a and b showed the morphology of scattered overlapped sheets with a moderate size of 12.42 nm. Furthermore, Fig. S10 a and b, respectively, show the EDX charting of L2 and C2.

The EDX plotting presented the attendance of nitrogen, carbon, oxygen and sulphur as the basic construction elements in L2, and nitrogen, carbon, oxygen, sulphur, chlorine and cerium as the basic construction elements in the distinct particles for C2. Furthermore, the EDX charting supported our explanation, and these findings agreed with those attained via elemental analysis as well as those estimated theoretically for L2; C, 47.81; N, 20.27; O, 5.78; and S, 23.20; found: C, 48.00; N, 20.67; O, 8.21; and S, 23.12 (Table S2); and for C2; C, 38.54; N, 16.05; O, 7.33; S, 14.69; Cl, 4.06; and Ce, 16.06; found: C, 38.83; N, 16.01; O, 11.12; S, 14.06; Cl, 3.98; and Ce, 16.00 (Table S3).

##### TEM

**Fig. 2:**
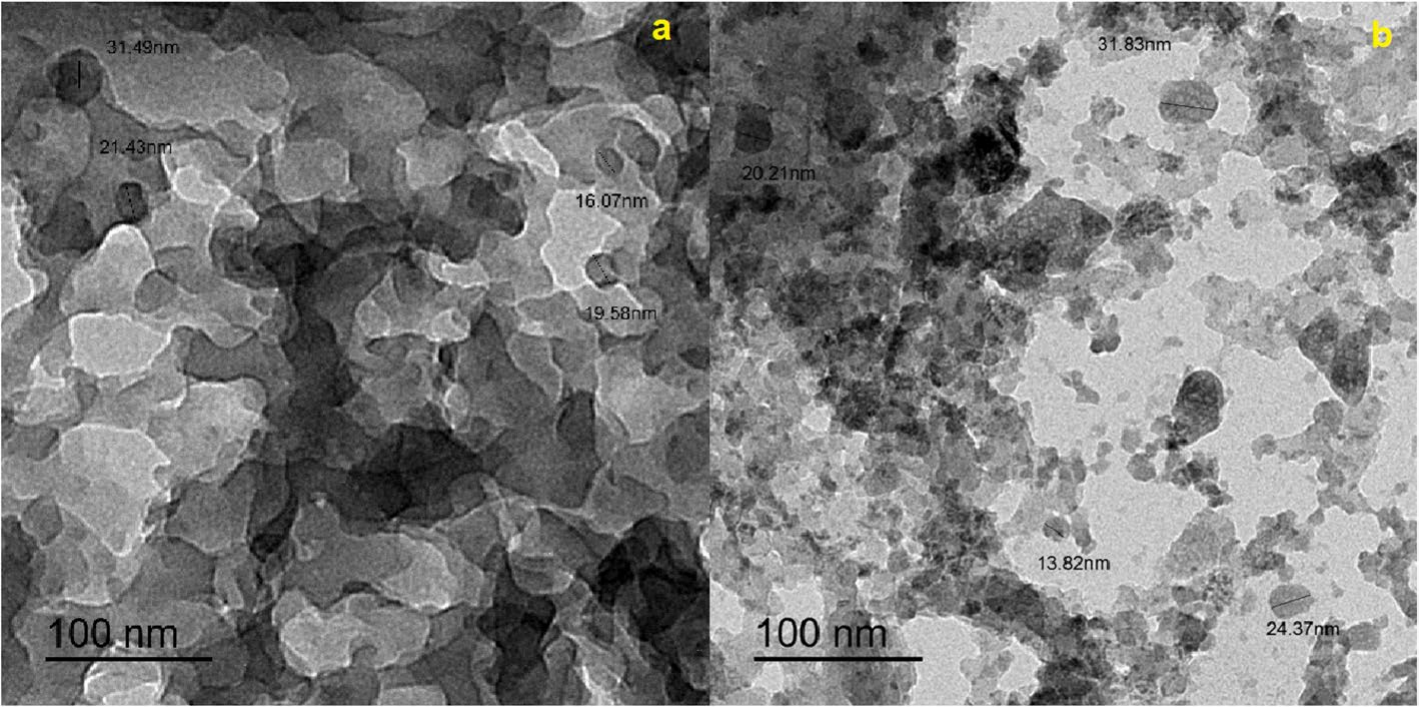
TEM image of: [**a**] Schiff base L2, and [**b**] Ce(III)-complex **C**2

Images from a transmission electron microscope (TEM) have been provided for L2 and C2 in Figs. 2a and b, correspondingly. The Ce-complex C2 morphology was shown in Fig. 2b as dispersed nanostructure particles, as opposed to the ligand L2 which was distributed as overlapping sheets in Fig. 2a. The findings obtained using the TEM modality was well-aligned and quite comparable with the data obtained using the FE-SEM.

#### Molecular docking

**Fig. 3:**
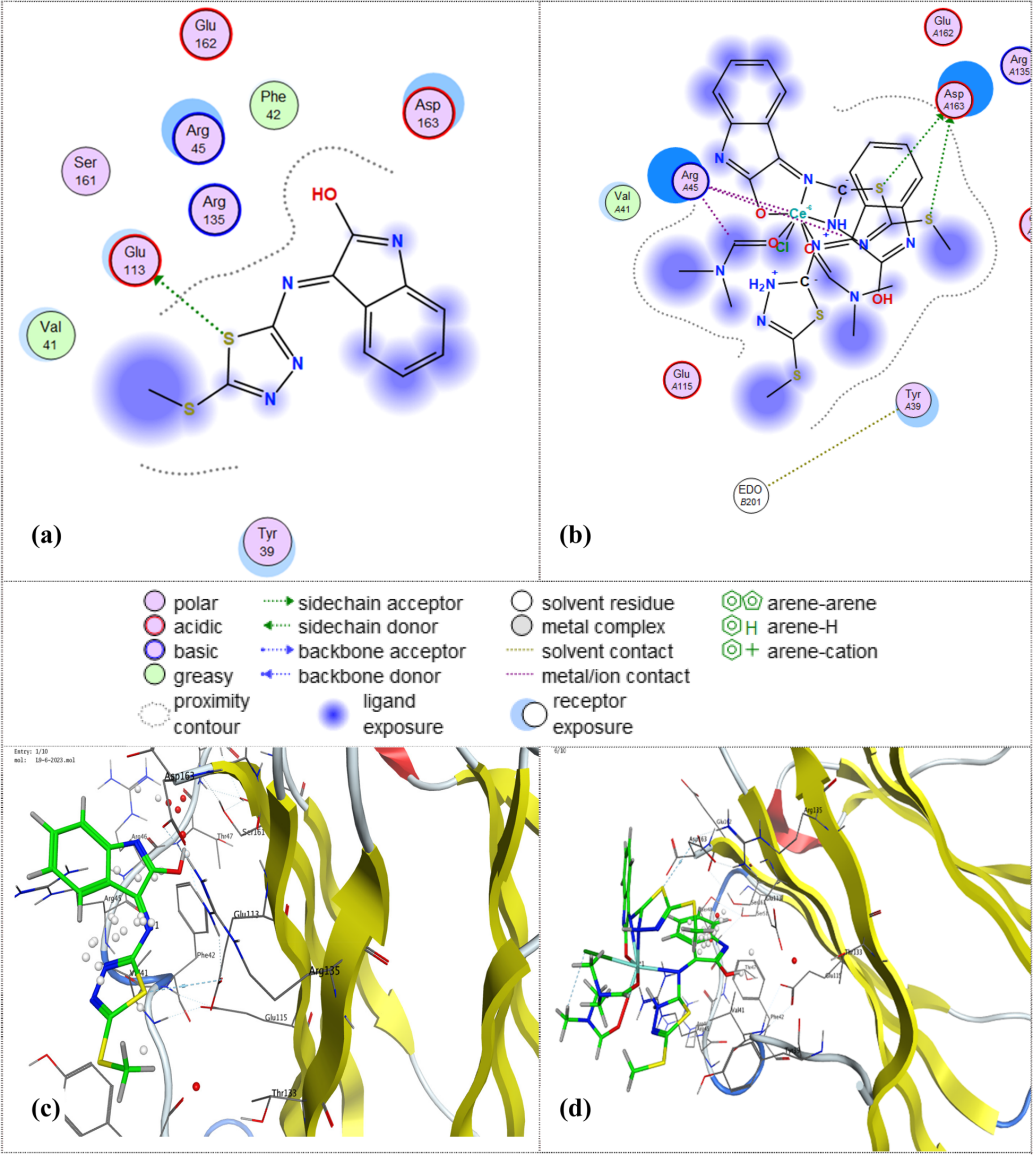
The 2D and 3D docking interaction of the ligand and its metal chelate with protein (protein data bank [PDB] code = 5OD4)

Molecular docking empowers us to comprehend the potential ways in which molecules may interact with one another and the attraction strengths of potent medications used for treatment purposes, to showcase the efficacy of newly developed compounds in exerting their desired biological effects on a specific target, 60 shortly we aim to demonstrate their remarkable bioactivity. Figure 3 illustrates the intricate and dynamic interplay in both three-dimensional (3D) and two-dimensional (2D) interactions between the ligand and its complex with 5OD4 protein downloaded from protein data bank (PDB). As per the analysis of molecular docking and interactions with proteins (ID: 5OD4), it has been observed that there are significant binding interactions occurring between the protein molecules. These interactions are crucial in determining the functional characteristics of these molecules. Ligand formed acidic side chain donner with the Glu 113 amino acid residue as shown in Fig. 3. It is worth mentioning that the unbound ligand exhibited a significantly low binding energy score (S = -4.7743 kcal/mol) and a high root mean square deviation in terms of atomic location (RMSD = 2.1349 kcal/mol). However, the complex formed five attractive bonds, two of them is an acidic sidechain donner with Asp A163, and the remained three bonds were metal/ion contact with Arg A45 (S = -28.7924 kcal/mol) and (RMSD = 3.292). These results summed up that the complex shows better docking score than the ligand.

#### The antimicrobial activity study

When categorizing antibacterial activity as either (Gram-negative) or (Gram-positive), a higher number of drugs typically exhibit effectiveness against (Gram-positive) bacteria compared to (Gram-negative) bacteria [54]. However, in this study, the titled compounds demonstrate a degree of activity against both bacterial types, suggesting potential broad-spectrum properties. The synthesized ligand and its complex underwent screening for antibacterial activity against Bacillus cereus (Gram-positive bacteria) and Staphylococcus aureus (Gram-negative bacteria), as well as antifungal activity against Aspergillus flavus (A. flavus). The antibacterial and antifungal activities of the ligand and its complexes are detailed in Table 2, evaluated using the diffusion agar technique.

**Table 2:** Antimicrobial activity of the ligand and its Ce^3+^ complex

compound	B.Cereus (gram + ve)	S. aureus (gram -ve)	A. flavus
Ligand	0.7	0.6	Inactive
Ce^3+^ complex	0.9	0.7	0.9
Ampicillin	1.3	1.5	––
Nystatin	––	––	1.3

The investigations revealed that the biological activity of the complex surpasses that of the ligand, although both are less effective than Ampicillin (antibacterial agent) and Nystatin (antifungal agent). Data presented in Table 2 indicates that the compounds in question exhibit higher effectiveness against Gram-positive bacteria when compared to Gram-negative bacteria, suggesting a correlation between antibacterial activity and the bacterial cell wall structure. Gram-positive bacteria, characterized by a thick cell wall comprising numerous layers of peptidoglycan and teichoic acids, are more susceptible to the studied compounds. On the other hand, when we consider Gram-negative bacteria, we observe that their cell wall is comparably thinner, composed of only a few layers of peptidoglycan enveloped within a lipid membrane that consists of lipopolysaccharides and lipoproteins, these entities experience comparatively lesser degrees of influence. The differences observed in the structure of cell walls play a significant role in determining the susceptibility of bacteria to antibacterial agents. These variations have a direct impact on how bacteria respond to treatment, highlighting the importance of understanding and recognizing the diversity within cell wall structures. By acknowledging these differences, we can better tailor our approach to effectively combat bacterial infections, As we know, several antibiotics are developed to specifically combat bacteria, but they might not always be effective against Gram-negative pathogens [54].

## Conclusions

New trivalent Ce^3+^ chelates with (E)-2-((5-methylthio)-1,3,4-thiadiazol-2-yl)imino)indolin-3-one (L2) derived from isatin and 2-amino-5-methylthio-1,3,4-thiadiazole were synthesized. The compounds were thoroughly analyzed using a combination of spectroscopic techniques and analytical methods in order to determine their structures. After conducting various analyses, including FT-IR spectral, XRD, TGA, DTG, elemental analysis, and molar conductance measurements it has been confirmed that the metal complexes were formed in the bimetallic form (L:M ration is 2:1). The FT-IR spectral results indicated the L2 acts as a neutral bi-dentate coordinate site through ν(OH), (C–N) and azomethine nitrogen (C = N) atoms and the UV- spectrum was referred to intra ligand charge transfers (n–π* and π–π*) and ligand metal charge transfer transitions (LMCT). The obtained results pointed out that the ligand and its complex were less effective against the tested microorganisms when compared with the standard drugs. The study showed that the complex was found to be more effective compound than the ligand. Extensive studies has been conducted to examine the remarkable antibacterial and antifungal properties of both the ligand and its complexes. Moreover, an assessment of the compound's docking behavior was conducted. Using molecular docking analysis, it is observed that L2 engages in favorable interactions with the residues present in the active site. Additionally, L2 exhibits a promising dock score (-4.7743 kcal/mol), indicating its potential efficacy. Through the molecular docking interaction process, L2 exhibited meaningful interactions with the residues present in the active site [55–61]. This interaction is further supported by a high dock score with -4.7743 kcal/mol, indicating a favorable binding potential. Based on the analytical, spectroscopic and thermal data discussed above, the structure of the complex can be formulated as proposed.

### Supplementary Information

**Additional file 1:** **Fig. S1.** The FT-IR spectra of Schiff base L2, and Ce(III)-complex C2. **Fig. S2.** The electronic absorption spectra of Schiff base L2, and Ce(III)-complex C2. **Fig. S3.** The ^1^H-NMR spectrum of Schiff base L2. **Fig. S4.** The ^1^H-NMR spectrum of Ce(III)-complex C2. **Fig. S5.** The ^13^C-NMR spectrum of Schiff base L2. **Fig. S6.** The ^13^C-NMR spectrum of Ce(III)-complex C2. **Fig. S7.** The mass spectrum of Schiff base L2. **Scheme S1.** The proposed fragmentation Scheme of Schiff base L2. **Fig. S8.** The mass spectrum of Ce(III)-complex C2. **Scheme S2.** The proposed fragmentation Scheme of the Ce(III)-complex C2. **Fig. S9.** DTA and TGA curves of Ce(III)-Schiff base complex (C2). **Fig. S10.** EDX images: [a–b] of Schiff base L2 and Ce(III)-complex C2. **Table S1.** Decomposition steps with the temperature range and weight loss for Ce(III)-Schiff base complex (C2). **Table S2.** EDX analysis of Schiff base L2. **Table S3.** EDX analysis of the Ce(III)-complex C2.

## Data Availability

All data are included in the manuscript, tables, figures and supplementary materials.
