# Supplementary material for: A novel isatin Schiff based cerium complex: synthesis, characterization, antimicrobial activity and molecular docking studies
Source: BMC Genomics. 2024 Feb 8;25:162. doi: 10.1186/s12864-024-10037-3 (PMC10854109; doi:10.1186/s12864-024-10037-3)
Supplement: Supplementary file 1 — Additional file 1: Fig. S1. The FT-IR spectra of Schiff base L2, and Ce(III)-complex C2. Fig. S2. The electronic absorption spectra of Schiff base L2, and Ce(III)-complex C2. Fig. S3. The 1H-NMR spectrum of Schiff base L2. Fig. S4. The 1H-NMR spectrum of Ce(III)-complex C2. Fig. S5. The 13C-NMR spectrum of Schiff base L2. Fig. S6. The 13C-NMR spectrum of Ce(III)-complex C2. Fig. S7. The mass spectrum of Schiff base L2. Scheme S1. The proposed fragmentation Scheme of Schiff base L2. Fig. S8. The mass spectrum of Ce(III)-complex C2. Scheme S2. The proposed fragmentation Scheme of the Ce(III)-complex C2. Fig. S9. DTA and TGA curves of Ce(III)-Schiff base complex (C2). Fig. S10. EDX images: [a–b] of Schiff base L2 and Ce(III)-complex C2. Table S1. Decomposition steps with the temperature range and weight loss for Ce(III)-Schiff base complex (C2). Table S2. EDX analysis of Schiff base L2. Table S3. EDX analysis of the Ce(III)-complex C2. [file 12864_2024_10037_MOESM1_ESM.docx]

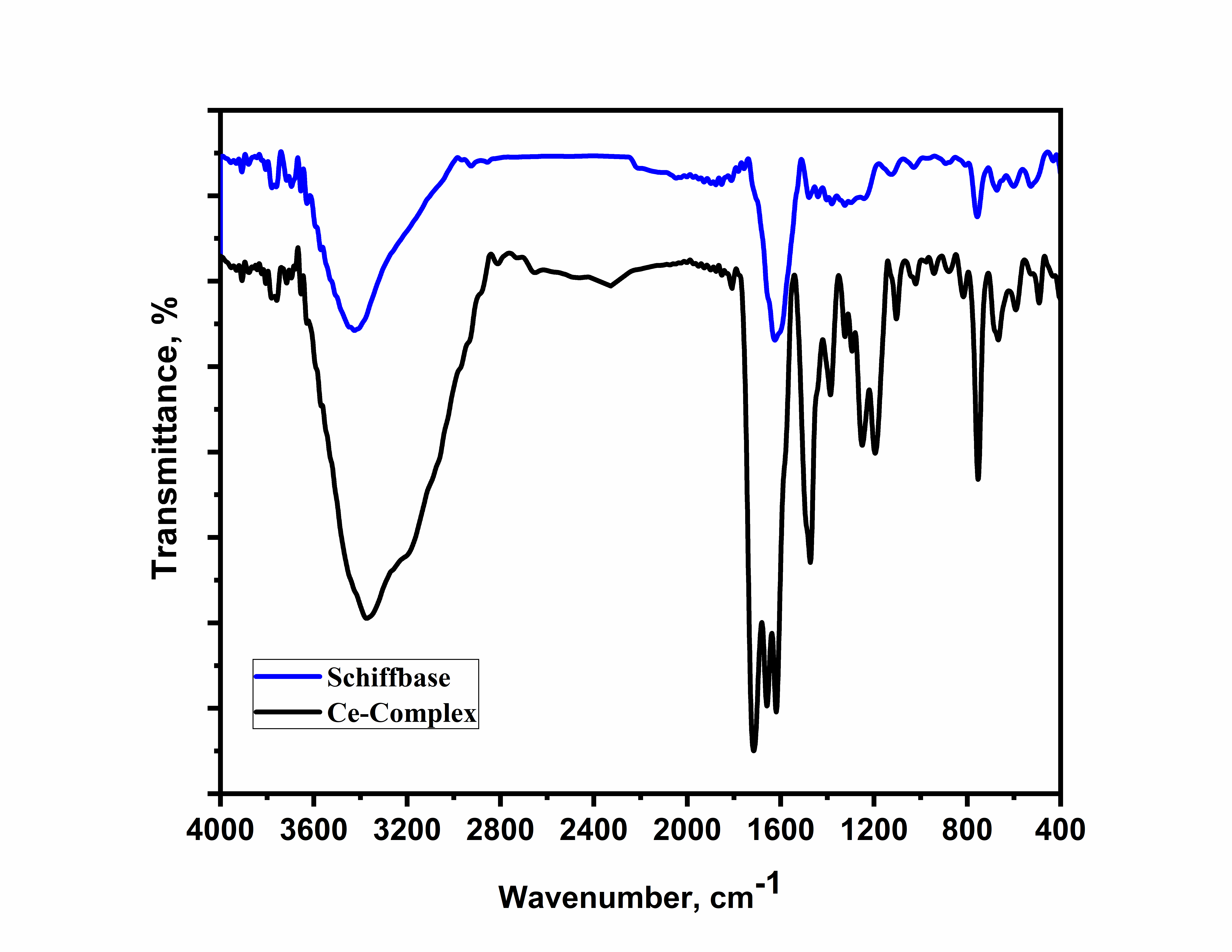


**Fig. S1:** The FT-IR spectra of Schiff base **L2**, and Ce(III)-complex **C2**.


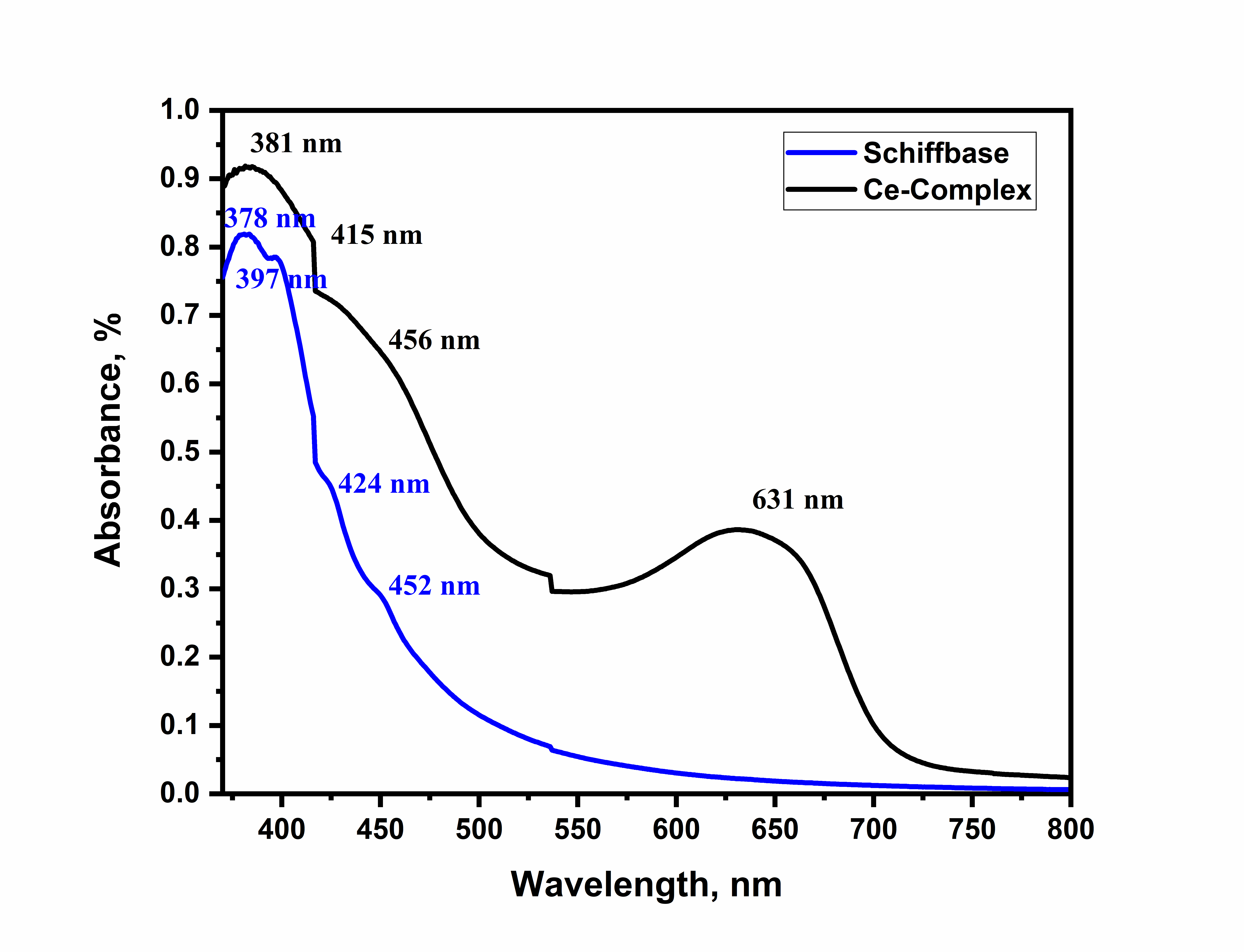


**Fig. S2:** The electronic absorption spectra of Schiff base **L2**, and Ce(III)-complex **C2**.


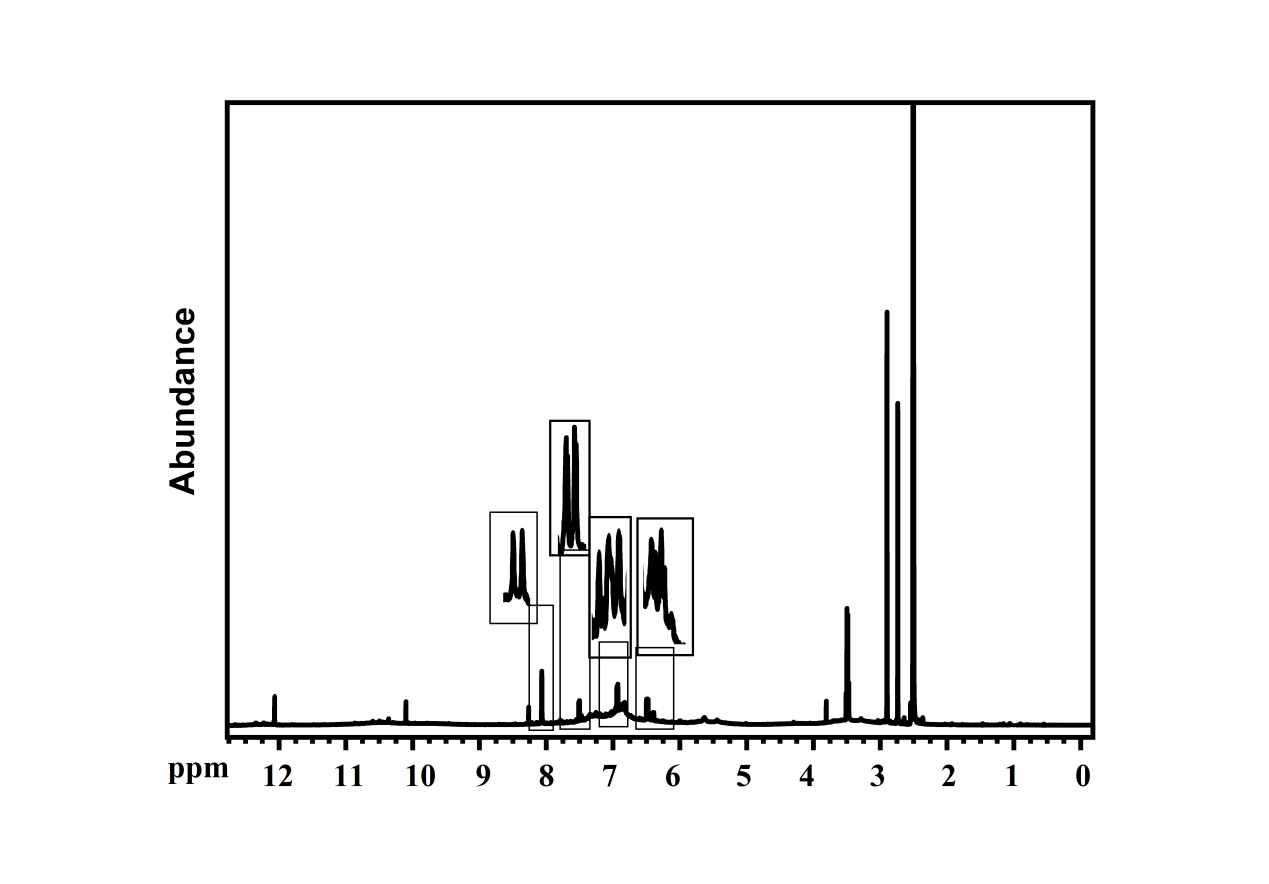


**Fig. S3:** The ^1^H-NMR spectrum of Schiff base **L2.**


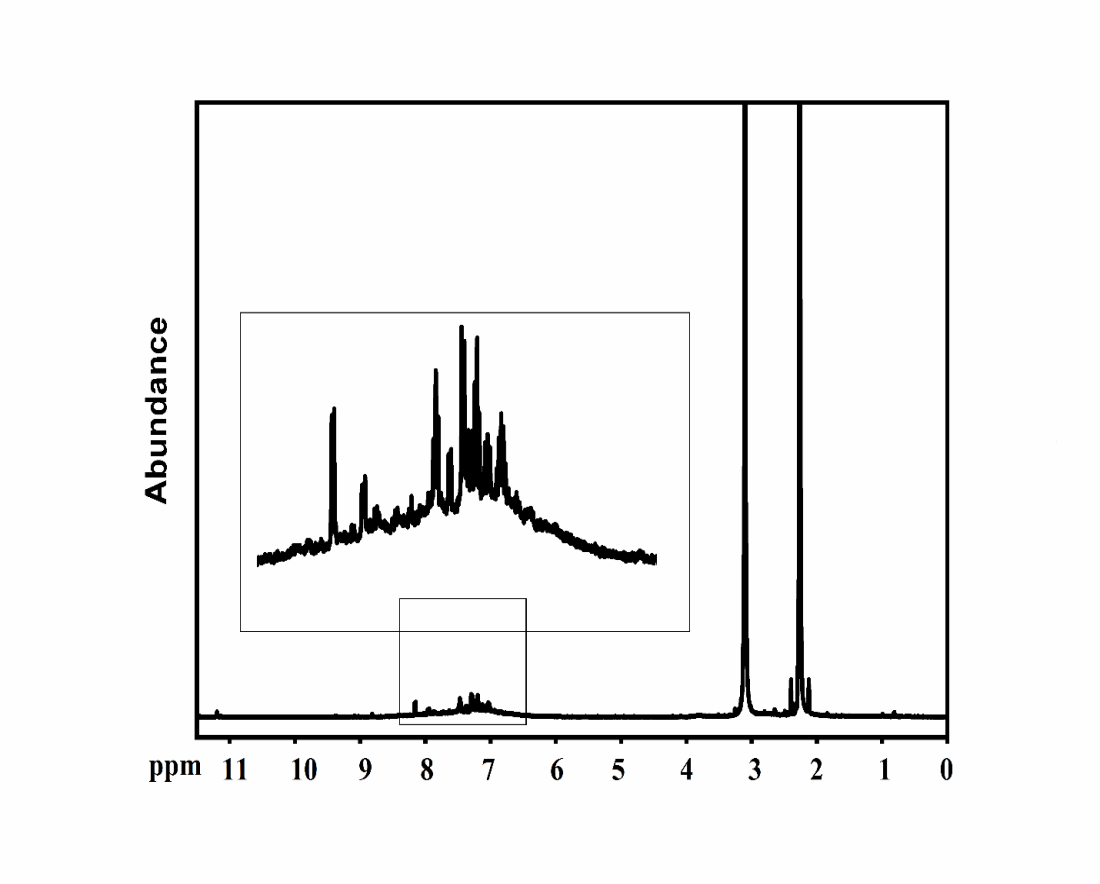


**Fig. S4:** The ^1^H-NMR spectrum of Ce(III)-complex **C2**.


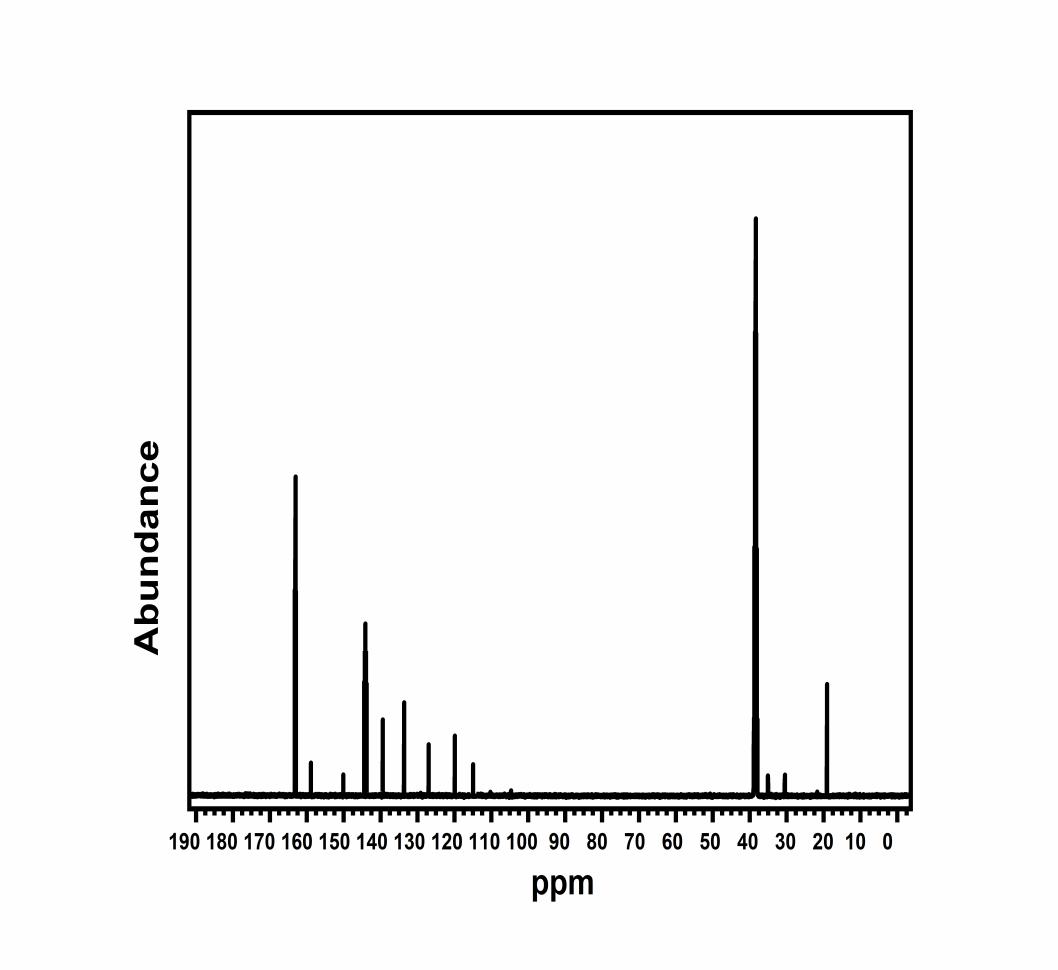


**Fig. S5:** The ^13^C-NMR spectrum of Schiff base **L2**.


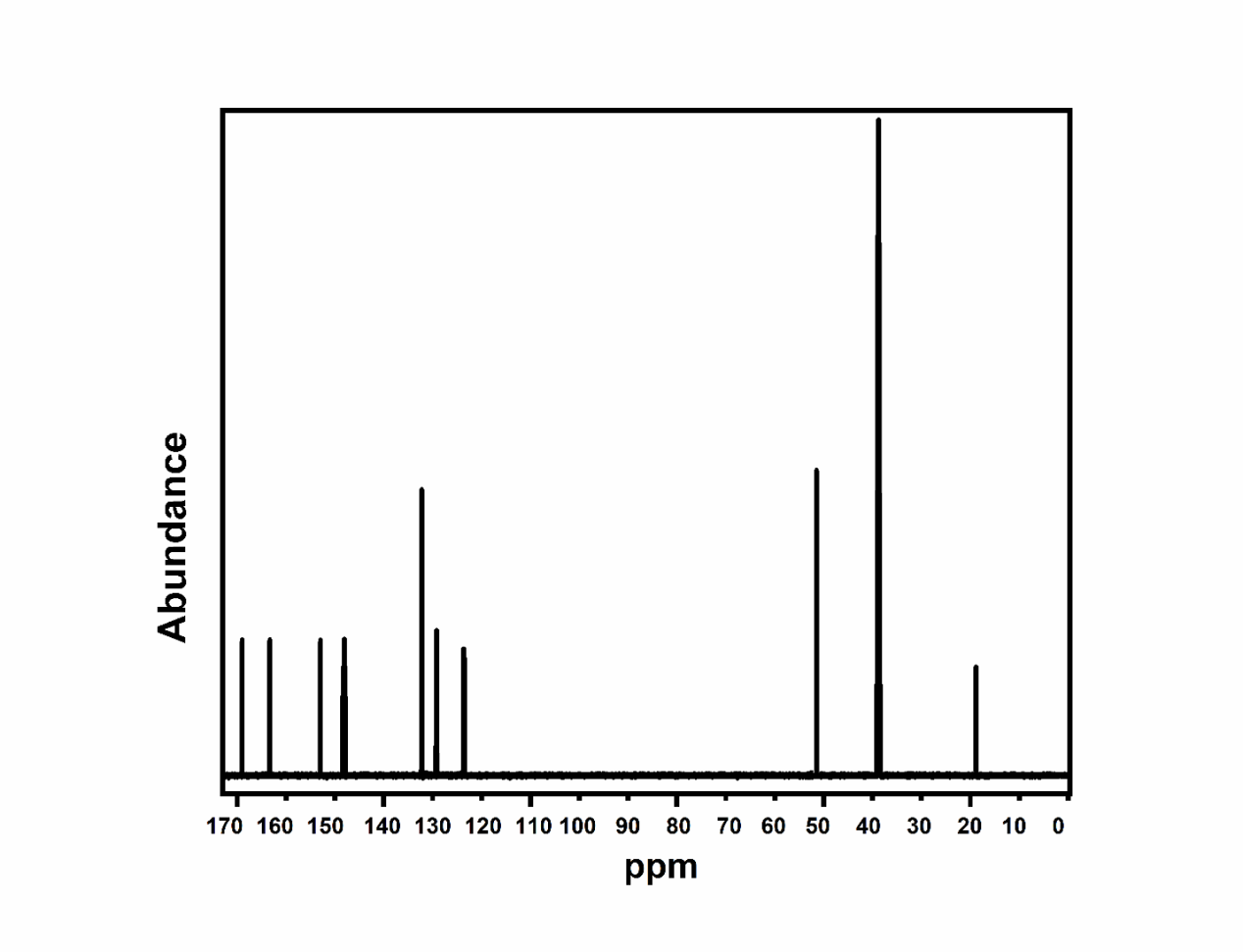


**Fig. S6:** The ^13^C-NMR spectrum of Ce(III)-complex **C2**.


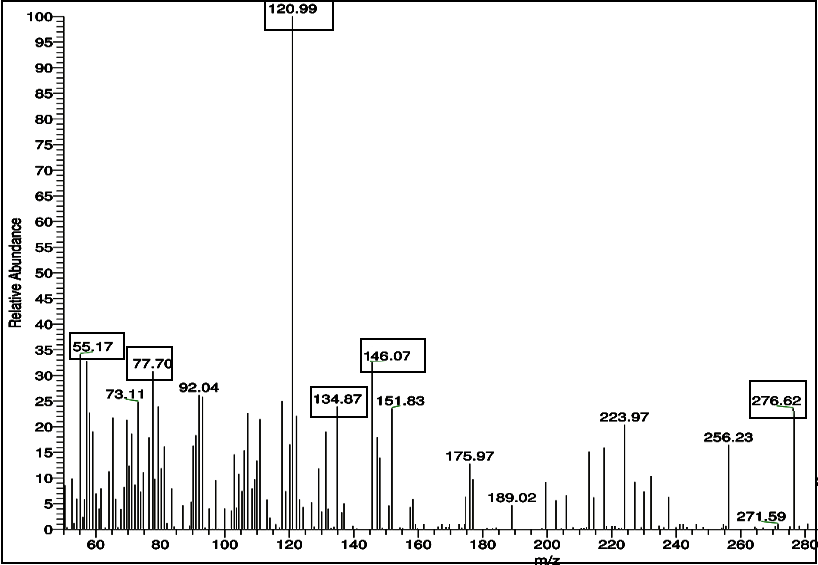


**Fig. S7:** The mass spectrum of Schiff base **L2**.


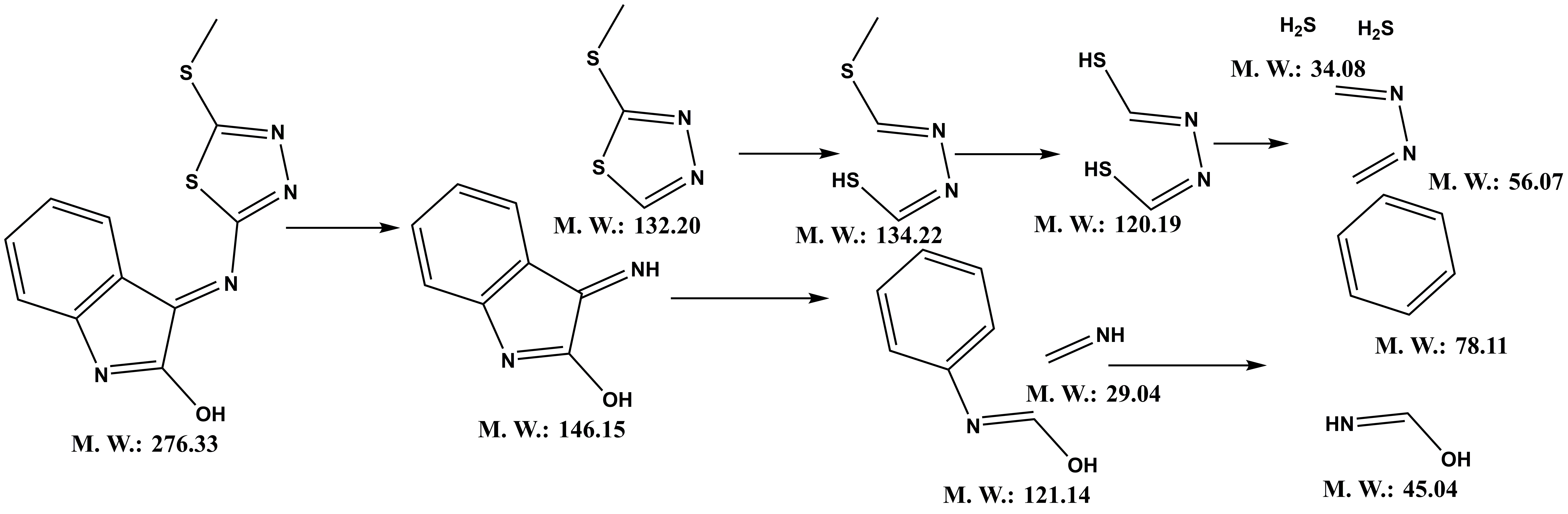


**Scheme S1:** The proposed fragmentation Scheme of Schiff base **L2**.


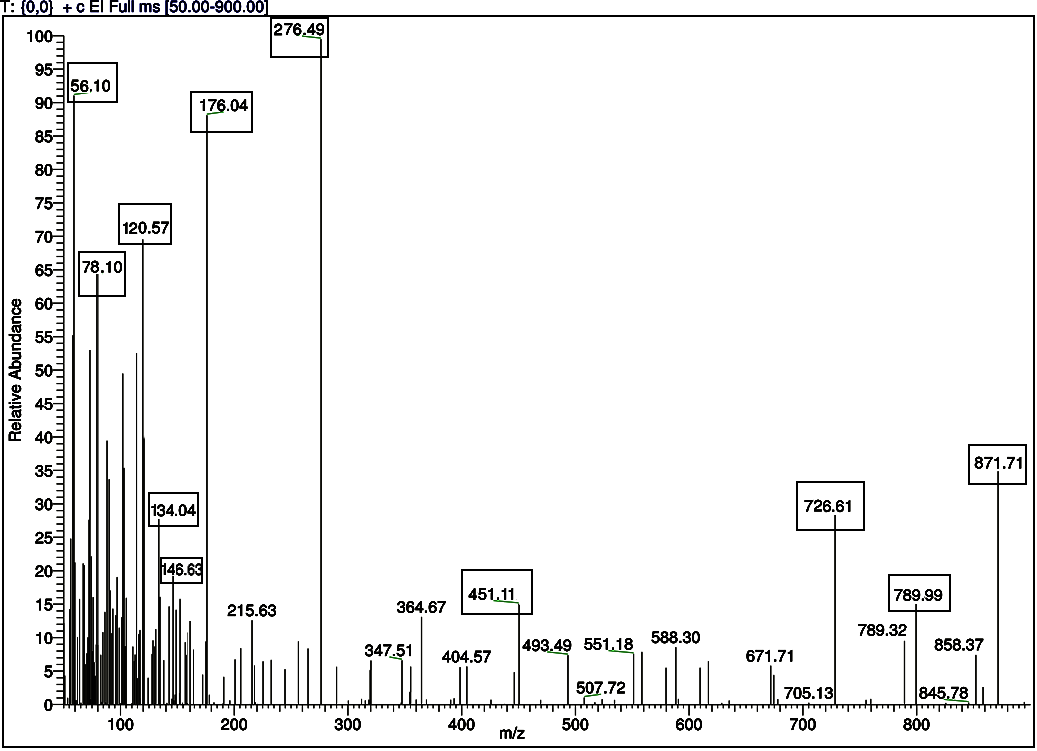


**Fig. S8:** The mass spectrum of Ce(III)-complex **C2**.





**Scheme S2:** The proposed fragmentation Scheme of the Ce(III)-complex **C2**.

**Fig. S9**: DTA and TGA curves of Ce(III)-Schiff base complex (**C2**).


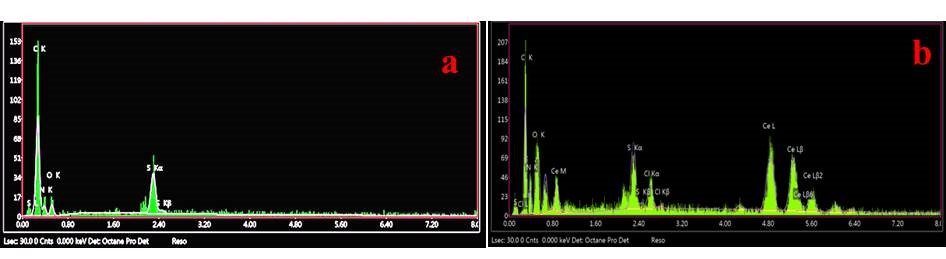


Fig. S10: EDX images: [a–b] of Schiff base L2 and Ce(III)-complex C2.

**Table S1**: Decomposition steps with the temperature range and weight loss for Ce(III)-Schiff base complex (**C2**).

| **Complex** | **Temp.**  **Range, °C** | **Removed species** | **Wt. Loss** | |
| --- | --- | --- | --- | --- |
|  |  |  | Found% | Calc.% |
| Cerium(III)-Schiff base complex **(C2)** | 28.81-139.81 | -2 DMF | 16.4 | 16.5 |
|  | 139.81-438.78 | - C_11_H_8_N_4_S_2_O | 31.6 | 31.5 |
|  | 438.78-609.75 | - CeCl | 19.95 | 20.01 |
|  | 609.75-730.67 | - C_3_H_4_N_2_S_2_ | 15.08 | 15.02 |
|  | 730.67-921.17 | - CH_3_N  -CH_3_NO  - C_6_H_6_ | 3.02  5.25  8.69 | 3.17  5.1  8.7 |

**Table** **S2:** EDX analysis of Schiff base **L2**.

| Element | Theoretically calculated | EDX analysis | | | |
| --- | --- | --- | --- | --- | --- |
|  |  | Weight % | Atomic % | Net Int. | Error % |
| C | 47.81 | 48.00 | 53.43 | 41.52 | 3.81 |
| N | 20.27 | 20.67 | 19.72 | 4.41 | 4.27 |
| O | 5.78 | 8.21 | 10.68 | 5.21 | 12.8 |
| S | 23.20 | 23.12 | 16.17 | 24.3 | 8.29 |

**Table** **S3:** EDX analysis of the Ce(III)-complex **C2**.

| Element | Theoretically calculated | EDX analysis | | | |
| --- | --- | --- | --- | --- | --- |
|  |  | Weight % | Atomic % | Net Int. | Error % |
| C | 38.54 | 38.83 | 45.96 | 1.77 | 5.55 |
| N | 16.05 | 16.01 | 17.46 | 4.23 | 4.23 |
| O | 7.33 | 11.12 | 14.53 | 70.84 | 10.64 |
| S | 14.69 | 14.06 | 15.84 | 52.68 | 12.25 |
| Cl | 4.06 | 3.98 | 2.82 | 20.83 | 8.91 |
| Ce | 16.06 | 16.00 | 3.39 | 117.05 | 7.8 |
